# ReVita: home-based transcutaneous auricular vagus nerve stimulation for reducing pathological fatigue in chronic neurological and post-infectious conditions—study protocol for a randomized, double-blind, sham-controlled trial

**DOI:** 10.1186/s13063-026-09919-6

**Published:** 2026-08-13

**Authors:** Anni Richter, Maria Kuehne, Margarita Darna, Tino Zaehle

**Affiliations:** 1 Department of Medical Psychology, Medical Faculty, Otto von Guericke University, Magdeburg, Germany; 2 German Center for Mental Health (DZPG), Partner Site Halle-Jena-Magdeburg, Magdeburg, Germany; 3 Center for Intervention and Research on adaptive and maladaptive brain Circuits underlying mental health (C-I-R-C), Halle-Jena-Magdeburg, Magdeburg, Germany; 4 Department of Neurology, Section Neuropsychology, Otto von Guericke University, Magdeburg, Germany; 5 Center for Behavioural Brain Sciences (CBBS), Magdeburg, Germany

**Keywords:** Pathological fatigue, Transcutaneous auricular vagus nerve stimulation, TaVNS, Neuromodulation, Randomized controlled trial, Sham-controlled, Digital health monitoring, Wearable sensors, Home-based stimulation, Transdiagnostic approach

## Abstract

**Background:**

Pathological fatigue is one of the most prevalent and disabling symptoms across chronic neurological, post-infectious, and inflammatory diseases, yet effective treatments remain limited. Transcutaneous auricular vagus nerve stimulation (taVNS) is a safe, non-invasive neuromodulatory intervention that targets neuroimmune and autonomic pathways implicated in the development and persistence of fatigue. This randomized controlled trial investigates whether home-based taVNS can reduce fatigue severity in a transdiagnostic population.

**Methods:**

This study is a randomized, double-blind, sham-controlled, parallel-group clinical trial conducted at the University Hospital Magdeburg. Eligible adult participants (≥ 18 years) with clinically significant chronic fatigue from two diagnostic clusters (neurological disorders and post-infectious syndromes) will be enrolled and randomized (1:1) to receive either active taVNS or sham stimulation for 4 weeks. Stimulation is self-administered at home twice daily for 30 min. The primary outcome is change in fatigue severity from baseline to the end of the intervention, assessed using the Modified Fatigue Impact Scale (MFIS). Secondary outcomes include quality of life (WHOQOL-BREF), depressive symptoms (BDI-II), daytime sleepiness (ESS), cognitive performance (SDMT and TAP subtest alertness), neurophysiological markers (resting-state EEG and P50 sensory gating) and HRV indices (Garmin Vivosmart 5). Blinding integrity will be assessed at study end. Data will be analyzed according to an intention-to-treat framework. No interim analyses are planned. Adverse events will be monitored continuously.

**Discussion:**

This is to be the first transdiagnostic study to evaluate home-based taVNS as a potential therapeutic approach for pathological fatigue across multiple chronic disease groups. The combination of rigorous blinding, digital monitoring, and parallel assessment of clinical and physiological parameters allows for an in-depth evaluation of both treatment effectiveness and mechanistic correlates. If successful, taVNS could offer a scalable and accessible intervention for a large and underserved patient population.

**Trial registration:**

The trial was registered in the German Clinical Trials Register (DRKS) under the identification number DRKS00038292. Registered on December 01, 2025. URL: https://drks.de/search/de/trial/DRKS00038292.

## Introduction

### Background and rationale {9a}

Pathological fatigue is a pervasive and disabling symptom that substantially interferes with daily functioning. Unlike normal, activity-related tiredness, it is often disproportionate to exertion, unpredictable in onset, and not alleviated by rest [1]. Fatigue is a multidimensional construct encompassing physical, cognitive, and psychosocial components, which has motivated unified conceptual frameworks and transdiagnostic approaches in both research and treatment development. To enhance comparability across studies, fatigue is commonly distinguished into subjective and objective dimensions. Subjective fatigue comprises long-lasting trait components as well as short-term state fluctuations, whereas objective fatigue refers to measurable performance decline during sustained cognitive or motor tasks [1, 2].

Fatigue is highly prevalent across numerous chronic conditions and thus constitutes a transdiagnostic symptom being frequently reported in neurological disorders (review: [3]) such as multiple sclerosis (MS; meta-analysis: [4]) and Parkinson’s disease (PD; meta-analysis: [5]), as well as in post-infectious syndromes including myalgic encephalomyelitis/chronic fatigue syndrome (ME/CFS; meta-analysis: [6]) and long COVID (meta-analysis: [7]). Importantly, the severity of fatigue is often only weakly related to underlying disease burden, supporting the view that fatigue constitutes a partly independent syndrome emerging from interactions among biological, psychological, and social factors [8, 9].

Accumulating evidence suggests that shared pathophysiological mechanisms contribute to fatigue across disease entities. Neuroimaging studies consistently implicate dysfunction within cortico-striatal circuits, including the basal ganglia and prefrontal cortex, which play a central role in motivation, reward processing, and effort regulation (review: [10, 11]). In addition, chronic low-grade inflammation and altered immune signaling involving pro-inflammatory cytokines such as interleukin-6 (IL-6) and tumor necrosis factor alpha (TNF-α) have been linked to fatigue across neurological and post infectious conditions (review: [12], meta-analysis: [13]). Autonomic dysregulation and alterations in central neuromodulatory systems, particularly monoaminergic and cholinergic pathways, further contribute to common fatigue phenotypes [11, 14, 15], providing converging mechanistic targets for intervention.

Despite the high prevalence and substantial impact of pathological fatigue, effective treatment options remain limited. Pharmacological interventions often show only modest efficacy and are frequently associated with relevant side effects (review: [16]). Consequently, increasing interest has focused on non-pharmacological approaches targeting shared neurobiological and immunological mechanisms. Recent clinical work has begun to systematically evaluate transcutaneous auricular vagus nerve stimulation (taVNS) in this context. Early human neurophysiological work demonstrated that transcutaneous stimulation of the auricular branch of the vagus nerve can elicit reproducible far-field evoked potentials originating from the brainstem, providing initial evidence for central engagement of this non-invasive approach [17]. Concurrent taVNS and functional magnetic resonance imaging (fMRI) studies demonstrate that stimulation of the auricular branch results in significant activation of central vagal afferent networks, including prefrontal and cingulate regions as well as basal ganglia, indicating that taVNS engages central autonomic and cognitive circuits (review: [18]). Recent protocols and pilot trials have targeted fatigue in long COVID and MS, demonstrating feasibility, safety, and preliminary indications of reduced mental fatigue in home-based settings ([19]; see also registered protocols: [20, 21]).

Taken together, pathological fatigue represents a major unmet clinical need. Its multidimensional and transdiagnostic nature highlights the importance of interventions that target shared neurobiological and immunological mechanisms rather than disease-specific features. In this context, taVNS emerges as a particularly promising candidate, as it directly modulates autonomic circuits and engages anti-inflammatory reflexes implicated across fatigue-related conditions. However, robust evidence from adequately powered, double-blind, transdiagnostic randomized controlled trials is still lacking. The present trial, therefore, aims to systematically evaluate the efficacy of taVNS within a transdiagnostic framework, with the goal of reducing pathological fatigue across a broad range of patient populations.

### Explanation for the choice of comparator {9b}

To rigorously test whether taVNS exerts effects beyond non-specific influences such as device handling, expectation, or daily symptom monitoring, a sham stimulation condition was chosen as the comparator. This approach allows for a high degree of experimental control consistent with international standards for non-invasive neuromodulation studies and ensures that observed effects can be attributed specifically to vagal activation rather than unspecific factors.

### Objectives {10}

The primary objective is to evaluate the efficacy of home-based taVNS in reducing subjective fatigue severity compared to sham stimulation in people with chronic pathological fatigue across neurological and post-infectious conditions.

#### Primary hypothesis

Active taVNS will lead to a greater reduction in Modified Fatigue Impact Scale (MFIS) total scores from baseline to post-intervention than sham stimulation.

#### Secondary hypotheses

Active taVNS will improve quality of life, sleep, cognitive performance, autonomic regulation, and reduce depressive symptoms compared with sham stimulation.

## Methods: patient and public involvement, and trial design

### Patient and public involvement {11}

Patients or members of the public were not involved in the design of this research. The study was developed by clinical researchers and methodologists with expertise in fatigue, neurostimulation, and clinical trial methodology. However, the study addresses a major unmet clinical need repeatedly voiced by patients with chronic pathological fatigue across conditions, who frequently report a lack of effective treatments and substantial impacts on functioning. These perspectives, documented in prior qualitative research and routine clinical care at the University Medical Center Magdeburg, informed the selection of outcomes, particularly subjective fatigue severity and quality of life. In addition, participant perspectives on feasibility, acceptability, and integration of the intervention into daily life will be systematically assessed using post-intervention questionnaires. While this does not constitute patient involvement in the design phase, it ensures that patient experiences are explicitly captured and will inform the interpretation and future implementation of the intervention. Following trial completion, a lay summary will be published on the study website and provided to interested participants in accessible language.

### Trial design {12}

The study is a randomized, double-blind, sham-controlled, parallel-group clinical trial with a transdiagnostic framework. Participants will be randomly allocated in a 1:1 ratio to active taVNS or sham stimulation for 4 weeks of home-based treatment. Two clinical populations will be included: neurological (i.e., MS, PD) and post-infectious (i.e., long COVID, ME/CFS). A follow-up assessment will occur 4 weeks post-intervention.

## Methods: participants, interventions, and outcomes

### Trial setting {13}

The study will be conducted at the University Medical Center Magdeburg, Germany, with recruitment through collaborating clinical departments (e.g., Neurology), outpatient neurology clinics, patient organizations, and social media channels (Table 1). Baseline and follow-up assessments will be performed on-site at the study center, whereas the intervention will be conducted in participants’ home environment using portable stimulation devices and a digital monitoring platform (*Labfront*, https://www.labfront.com, [22]). Remote supervision will be provided throughout the intervention phase to ensure treatment adherence, safety, and data integrity.

**Table 1:** Characteristics of the people who are needed for the trial

Characteristic	The people we would expect to see included
Age	Adults aged ≥ 18 years, expected mean in mid-40 s. Exact mean/median age and variability (range or SD) will be reported at baseline
Sex	Participants describing their sex as Male, Female, or Intersex, according to self-report
Gender	Participants identifying as men, women, or other gender identities (e.g., non-binary)
Race, ethnicity, and ancestry	Categories as self-reported by participants, likely reflecting the local population in Germany (e.g., White/European, Middle Eastern, Asian, African, or other categories as provided by participants). These will be documented exactly as reported
Socioeconomic status	Socioeconomic indicators if available, such as educational attainment and employment status reported as provided by participants
Geographic location	Individuals living in Germany, primarily in the recruitment region surrounding the University Medical Center Magdeburg
Other characteristics relevant to the trial	- Diagnosed with one of the eligible conditions (MS, PD, post-viral fatigue) - Sufficient German language proficiency - Able and willing to use a smartphone and wearable device - Experiencing clinically relevant fatigue for ≥ 3 months

### Eligibility criteria for participants {14a}

Eligibility criteria were defined to ensure a clinically relevant level of fatigue, the safety of participants, and the feasibility of home-based taVNS.

#### Inclusion criteria

- Minimum age 18 years
- Ability to provide informed consent
- Sufficient German language proficiency to complete questionnaires and follow instructions
- Clinically relevant fatigue (≥ 3 months duration and above cut-off on the fatigue severity scale, FSS ≥ 4 [23])
- Belonging to one of the target patient groups: Neurological: Confirmed diagnosis of MS or PD; Post-infectious: e.g., long COVID or ME/CFS (fatigue developed in temporal association with a documented viral infection)
- Ownership and ability to use an internet-enabled smartphone and wearable or have a person (relative or caregiver) to support them
- Ability and willingness to attend on-site study visits at the University Medical Center Magdeburg and to participate in all required study procedures

#### Exclusion criteria

- Presence of implanted electronic devices (e.g., pacemaker, cochlear implant, deep brain stimulation)
- History of epilepsy
- Current substance use disorder or substance use disorder within the past 2 years
- Acute psychiatric disorders (e.g., psychosis, bipolar disorder) or suicidal ideation within the last 6 months
- Diagnosis of chronic migraine or other primary headache disorder with clinically relevant symptoms
- History of neurosurgical or intracranial surgery
- Pregnancy or breastfeeding
- Unstable medication within the last 3 months
- Unstable medical conditions (e.g., acute MS relapse within 3 months)
- Clinically significant cardiac arrhythmia
- Mild to moderate dementia (below cutoff in the Montreal Cognitive Assessment’s auditory items, MoCA-22 < 13 [24])
- Current participation in another interventional trial

Additional exclusion criteria may include known hypersensitivity to electrode materials or inability to comply with study procedures.

### Eligibility criteria for sites and those delivering interventions {14b}

The intervention will be delivered by experienced researchers at the University Medical Center Magdeburg who are trained in non-invasive neurostimulation and the study-specific protocol.

### Who will take informed consent? {32a}

Written informed consent will be obtained from all participants prior to any study-related procedures. Consent will be taken by trained study personnel from the University Medical Center Magdeburg who have completed Good Clinical Practice (GCP) training.

Potential participants will receive verbal and written information about the study aims, procedures, potential risks and benefits, data handling, and their rights, including the right to withdraw at any time without providing a reason and without any consequences for their medical care. Adequate time will be provided to consider participation and to ask questions before signing the consent form. The consent discussion will take place in a private setting at the study site to ensure confidentiality. A copy of the signed consent form will be provided to each participant, and the original will be stored in the investigator site file in accordance with data protection regulations.

### Additional consent provisions for collection and use of participant data and biological specimens {32b}

Not applicable. No biological specimens will be collected as part of this trial.

## Intervention and comparator

### Intervention and comparator description {15a}

Participants will be randomized to receive either active or sham taVNS for a period of 4 weeks. Stimulation will be applied via surface electrodes placed at the left cymba conchae, targeting afferent fibers of the auricular branch of the vagus nerve. Left-sided stimulation is used to avoid potential interaction with right-sided vagal cardiac innervation.

Stimulation will be delivered using CE-certified devices (tVNS Technologies GmbH, Germany). Parameters will be set to a frequency of 25 Hz, a pulse width of 250 μs, and an individually adjusted current intensity (perceptible but comfortable), delivered in an ON/OFF pattern (28 s ON/32 s OFF).

Participants in the sham group will undergo an identical procedure with stimulation devices that are externally indistinguishable from those used in the active group. In the sham condition, no effective electrical stimulation of vagal afferents is applied.

Participants will use the Labfront mobile application on their personal smartphones, which delivers automated session reminders and collects daily self-report measures (sleep, fatigue, well-being, side effects). A Garmin Vivosmart 5 tracker will collect continuous physiological data (heart rate, physical activity/actigraphy). Data will be transferred in pseudonymized form to secure servers and subsequently stored on encrypted systems at the University Medical Center Magdeburg.

### Criteria for discontinuing or modifying allocated intervention/comparator {15b}

Participants may discontinue or temporarily interrupt the allocated intervention (active or sham taVNS) under the following circumstances.

#### Adverse events (AEs)

If participants experience AEs possibly related to stimulation (e.g., persistent skin irritation, dizziness, headache, or discomfort at the stimulation site) that persist despite adjustment of stimulation intensity or electrode contact, stimulation will be paused or discontinued.

#### Serious adverse events (SAEs)

In the case of an SAE where continuation of the intervention poses potential risk, the intervention will be stopped. Emergency unblinding will occur only if essential for clinical decision-making.

#### Participant request

Participants may withdraw from the intervention and/or study at any time without providing a reason.

#### Investigator decision

The investigator may discontinue participation in the case of safety concerns, protocol deviation or non-adherence (e.g., new occurrence exclusion criteria, disease instability, non-compliance with study procedures). If participants fail to perform stimulation sessions as instructed on three consecutive days, the study team will contact the participant to assess reasons and provide support. If non-adherence persists, intervention discontinuation may follow, while outcome assessments will still be encouraged for intention-to-treat (ITT) analysis.

All discontinuations and modifications will be documented in the electronic case report form, including date, reason, and decision-making authority.

### Strategies to improve adherence to intervention/comparator {15c}

The following measures will be implemented to promote adherence.

#### Standardized training and supervised initiation

At baseline (T0), all participants will receive standardized instruction and supervised hands-on training by trained personnel on device setup, electrode placement, and troubleshooting. The first stimulation is conducted under investigator supervision to ensure adequate comprehension and safe application. Participants also receive a written instruction manual including safety guidance and frequently encountered issues for home administration. The intervention itself is structured as 2 × 30-min sessions daily and can be integrated into daily routines. Portable devices with intuitive interfaces aim to further minimize burden.

#### Digital reminders and monitoring

Daily automated reminders will be sent via the Labfront mobile application, prompting participants to perform their scheduled morning and evening stimulation sessions. The app also collects real-time adherence data (session stop times, survey completion), which are transmitted to the study team. Participants showing reduced compliance or irregular usage patterns will be contacted via app, phone or email to identify potential difficulties and provide individualized guidance or troubleshooting. Positive feedback and clear communication will emphasize the importance of adherence for the validity of study results and potential individual benefit.

Overall adherence will be quantified as the proportion of completed stimulation sessions relative to the total number of prescribed stimulation sessions. Adherence data will be included as a continuous covariate in exploratory analyses to assess potential dose–response relationships between taVNS use and treatment outcomes.

### Concomitant care permitted or prohibited during the trial {15d}

Participants may continue receiving standard medical care. Pharmacological and non-pharmacological treatments must be stable ≥ 3 months prior to baseline and expected to remain unchanged. Short-term symptomatic treatments as clinically indicated are allowed. All medications will be documented at T0 and reviewed at the post-intervention/follow-up assesments (T1/T2) for protocol deviations and potential confounders.

### Ancillary and post-trial care {34}

Given that taVNS is non-invasive and generally low risk, no routine ancillary medical care beyond standard clinical management is anticipated as part of the trial. Participants will continue to receive routine medical care for their underlying condition throughout the study.

In the case of an AE judged to be related to the study intervention, the study team will ensure prompt medical evaluation and arrange appropriate treatment per institutional clinical protocols. All AEs will be managed under the supervision of qualified investigators and documented in accordance with GCP and institutional reporting procedures.

Participants assigned to the sham stimulation group will be offered the opportunity to try active taVNS after completion of the study, if no contraindications or safety concerns are identified. This optional open-label phase provides equitable access to the active intervention as an ethical post-trial provision.

Participants will not receive monetary compensation. As a non-monetary token of appreciation, participants who complete the study (including follow-up) may retain the Garmin Vivosmart 5 device used during the trial.

### Outcomes {16}

#### Primary outcome

The primary outcome is the change in subjective fatigue severity from baseline (T0) to post-intervention (T1), measured by the Modified Fatigue Impact Scale (MFIS; [25, 26]). The 21-item scale accounts for the physical, cognitive, and psychosocial dimensions of fatigue, and the overall score ranges from 0 to 84, with higher scores representing higher levels of fatigue. A larger reduction in MFIS score from T0 to T1 in the active versus sham group will be interpreted as evidence of treatment efficacy.

#### Secondary outcomes

Secondary outcomes include self-reported, cognitive, and neurophysiological measures to comprehensively assess fatigue-related domains:

- Quality of life (World Health Organization Quality of Life - brief version, WHOQOL-BREF; review: [27])
- Depressive symptoms (Beck Depression Inventory, BDI-II, [28])
- Daytime sleepiness (Epworth Sleepiness Scale, ESS, [29])
- Cognitive performance (electronic Symbol Digit Modalities Test, eSDMT, [30–32]; alertness subtest of the Test of Attentional Performance, TAP, [33, 34])
- Neurophysiological markers (resting-state electroencephalography, rsEEG, [35]; P50 sensory gating [36])

All secondary outcomes will be assessed at T0 and T1. To investigate persistence of effects or potential delayed responses, follow-up changes at T2 will be analyzed for selected secondary outcomes (questionnaires: WHOQOL-BREF, BDI-II, ESS).

#### Exploratory outcomes

Exploratory outcomes will focus on identifying potential moderators and mediators of treatment response and on characterizing physiological and experiential aspects of fatigue. These analyses will include physiological measures such as autonomic regulation derived from heart rate variability (HRV) and activity data obtained through continuous actigraphy (Garmin Vivosmart 5), as well as subjective sleep quality and daily well-being captured through app-based self-reports throughout the intervention period. Potential moderators such as diagnostic group (neurological vs. post-infectious), adherence metrics (e.g., session completion rate, average stimulation intensity) will also be examined. In addition, usability and acceptance of the home-based taVNS application will be evaluated at the post-intervention assessment (T1) using a standardized evaluation questionnaire.

### Harms {17}

AEs and device-related side effects (e.g., skin irritation, headache, dizziness) will be systematically recorded throughout the intervention phase using standardized daily self-reports and a structured questionnaire at T1 (Comfort Rating Questionnaire, CRQ; [37]). All AEs will be categorized by severity, expectedness, and causal relatedness to the intervention. SAEs will be reported to the sponsor within 24 h.

### Participant timeline {18}

**Fig. 1:**
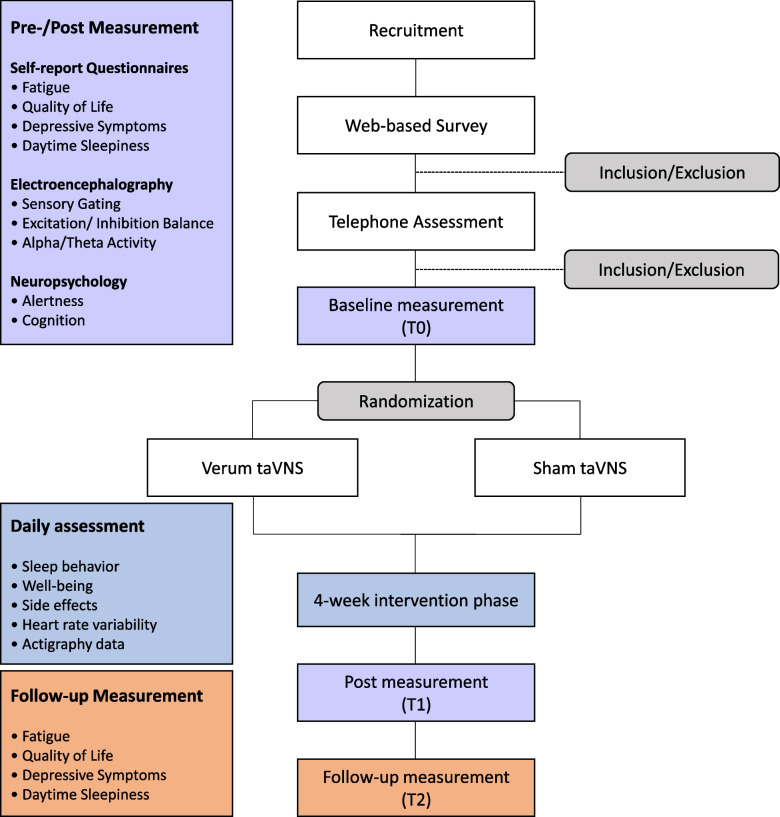
Study design and intervention timeline

The study comprises a baseline visit (T0), a 4-week home-based intervention phase, and a post-intervention assessment (T1; see Table 2 and Fig. 1). A follow-up assessment (T2) will be conducted 4 weeks after T1 to evaluate the sustainability of treatment effects.

**Table 2:** Schedule of enrollment, interventions, and assessments

Study period					
**Timepoint**	**Enrollment**	**Baseline (T0)**	**Intervention** **(4 weeks)**	**Postintervention (T1)**	**Follow-up (T2)**
**Enrollment**					
Eligibility screen	X				
Informed consent		X			
Randomization		X			
Device instruction and first supervised stimulation		X			
Distribution of fitness tracker and app introduction		X			
**Intervention**					
Active taVNS (2 × 30 min per day)			X		
Sham taVNS (2 × 30 min per day)			X		
**Assessments**					
Remote screenings (FSS, MoCA-22)	X				
Questionnaires (MFIS, WHOQOL-BREF, BDI-II, ESS)		X		X	X
Electroencephalography (Resting State, P50)		X		X	
Neuropsychology (SDMT, TAP Alertness)		X		X	
App-based self-reports (sleep, well-being, side effects)			X		
Fitness tracker (HRV and actigraphy)			X		

*taVNS* transcutaneous auricular vagus nerve stimulation, *FSS* Fatigue Severity Scale, *MoCA-22* Montreal Cognitive Assessment’s auditory items, *MFIS* Modified Fatigue Impact Scale, *WHOQOL-BREF* World Health Organization Quality of Life, *BDI-II* Beck Depression Inventory, *ESS* Epworth Sleepiness Scale, *SDMT* Symbol Digit Modalities Test, *TAP* tonic/phasic Alertness Task, *HRV *heart rate variability

### Sample size {19}

The sample size was calculated to detect a medium effect size (*f* = 0.25 according to Cohen) for the difference in change of MFIS total score between the active and sham taVNS groups from baseline (T0) to post-intervention (T1). Assuming a two-tailed α = 0.05 and power (1–*β*) = 0.80, a total of *N* = 128 participants (*n* = 64 per group) are required to detect this effect employing an *F*-test for repeated-measures (within–between interaction). To account for an anticipated drop-out rate of approximately 15%, we aim to enroll *N* = 150 participants in total. The final sample will be stratified across diagnostic categories (neurological, post-infectious) to ensure sufficient representation and enable exploratory subgroup analyses. Power calculations were performed using G*Power version 3.1 ([38]; Heinrich-Heine University Düsseldorf, Germany).

### Recruitment {20}

Participants will be recruited at the University Medical Center Magdeburg, Germany, through collaborating clinical departments (e.g., Neurology), as well as via patient organizations, outpatient clinics, and online advertisements (e.g., institutional websites and social media channels). Recruitment information was reviewed and approved by the Ethics Committee prior to dissemination.

Potential participants will first complete an online pre-screening, followed by a telephone screening interview to assess preliminary eligibility according to the predefined inclusion and exclusion criteria. Those meeting the criteria will be invited to an on-site baseline visit (T0), where written informed consent will be obtained before any study-related procedures.

Recruitment will focus on individuals experiencing clinically relevant, persistent fatigue in the context of chronic neurological or post-infectious disorders. This transdiagnostic recruitment strategy ensures the inclusion of a broad and representative sample of persons affected by pathological fatigue, without emphasizing disease-specific comparisons.

Participants will not receive financial incentives, but as a token of appreciation, participants may keep the fitness tracker used during the study after completing the follow-up assessment. Recruitment will continue until the target sample size is achieved. Recruitment is expected to take approximately 24 months.

## Assignment of interventions: randomization

### Sequence generation: who will generate the sequence {21a}

Randomization will be performed centrally by a study team member not involved in outcome assessments.

### Sequence generation: type of randomization {21b}

Participants will be stratified according to diagnostic categories (neurological and post-infectious). Within each stratum, participants will be randomly allocated to the intervention groups using block randomization with variable block sizes of 4 and 6. This approach ensures a balanced distribution of participants across active and sham groups while minimizing predictability of allocation.

### Allocation concealment mechanism {22}

Randomization codes will be generated using a computerized randomization algorithm and implemented through the REDCap Randomization Module [39, 40]. Allocation sequences will be stored within REDCap’s secure, role-restricted randomization table, which is inaccessible to investigators involved in participant assessment or data analysis. Study personnel will assign participants via REDCap using an automated, concealed allocation process that reveals group assignment only after all required baseline data have been entered and eligibility has been confirmed. All study staff conducting outcome assessments and analyses will remain blinded to treatment allocation until the final data lock.

### Implementation {23}

Participants will be enrolled and consented by trained study staff at the University Medical Center Magdeburg. Following eligibility confirmation, each participant will be assigned the next sequential study identification number (ID) by a non-assessing team member who selects the preprogrammed allocated device (active or sham). This process ensures concealment of allocation from both participants and investigators.

## Assignment of interventions: blinding

### Who will be blinded {24a}

This study employs a double-blind design to minimize bias and ensure the validity of results. Both participants and investigators involved in outcome assessment will be blinded to group allocation (active vs. sham taVNS).

#### Participants

All participants will receive stimulation using identical-appearing devices with the same interface, sounds, and operational procedures. They will not be informed of their group assignment or the specific stimulation parameters used.

#### Outcome assessors and study personnel

Investigators conducting clinical, neurophysiological, and cognitive assessments, as well as data analysis, will remain blinded to treatment allocation. Separate study staff members will handle randomization, device programming, and allocation concealment to prevent any unintentional unblinding. Statistical analyses will be performed on anonymized datasets labeled only with coded group identifiers (“Group A”/“Group B”) until the database is locked and primary analyses are finalized.

### How will blinding be achieved {24b}

Blinding will be maintained through the use of externally indistinguishable stimulation devices and standardized procedures for both the active and sham conditions.

#### Device appearance and operation

Both active and sham taVNS devices (tVNS Technologies GmbH, Germany) are identical in appearance, interface, sound, and handling. Each device will be preselected according to the randomization allocation by a study team member not involved in outcome assessment.

#### Participant instructions

Participants in both groups will receive identical instructions for electrode placement, device use, and stimulation schedule.

In the active taVNS group, stimulation intensity will be individually adjusted to a level just above the perceptual threshold, producing a mild, non-painful tingling sensation.

In the sham group, during instruction, stimulation intensity is set just below the perceptible threshold, so that no noticeable stimulation occurs. During intervention, no effective stimulation will be conducted while all other instructions and device handling procedures are identical for both groups.

Both groups will thus experience comparable device interaction and treatment expectations, supporting the credibility of the sham condition.

#### Investigator blinding

Investigators performing baseline, post-intervention, and follow-up assessments (T0, T1, T2) will have no access to device allocation codes. All outcome data will be recorded using participant study identification numbers (IDs) only.

#### Assessment of blinding integrity

At the end of the study (T2), both participants and outcome assessors will be asked to guess the assigned condition (“active,” “sham,” or “don’t know”) to evaluate the success of blinding.

Together, these measures ensure that participants and assessors remain effectively blinded throughout the intervention and follow-up phases, minimizing bias due to expectancy or observer effects.

### Procedure for unblinding if needed {24c}

Unblinding will occur only in the event of a SAE when knowledge of the allocation is necessary for clinical management.

## Data collection and management

### Plans for assessment and collection of outcomes {25a}

All study data will be collected according to a predefined schedule at three time points: baseline (T0), post-intervention (T1), and follow-up (T2). Baseline and post-intervention assessments will take place on-site at the University Medical Center Magdeburg, whereas the intervention phase and follow-up will be conducted at participants’ homes using digital monitoring tools.

#### On-site assessments

At baseline (T0) and post-intervention (T1), participants will complete a battery of validated questionnaires (MFIS, WHOQOL-BREF, BDI-II, ESS) and undergo neurophysiological (EEG recordings: P50 suppression, resting state) and neurocognitive testing (eSDMT, tonic/phasic Alertness Task).

#### Remote data collection

During the 4-week intervention phase, participants will use the Labfront mobile app for daily self-report measures on sleep quality, well-being, and potential side effects. Additionally, they will wear a Garmin Vivosmart 5 tracker to continuously record HRV and actigraphy-based activity levels. All data from the app and the tracker are automatically pseudonymized and encrypted upon upload to Labfront’s secure cloud infrastructure and subsequently transferred to the University Medical Center Magdeburg for storage and analysis.

### Plans to promote participant retention and complete follow-up {25b}

Participants will be allowed to retain the Garmin device after completing the follow-up assessments as a token of appreciation.

### Data management {26}

Data will be stored on a secure institutional server in accordance with EU General Data Protection Regulation (GDPR) standards. Each participant will be identified only by a unique study ID; no personally identifiable information will be used in analysis datasets. Access to the full database is restricted to authorized study personnel. To ensure data quality, checks will be implemented in the electronic database. EEG and physiological data will undergo standardized preprocessing pipelines (artifact correction, filtering, and quality control).

### Confidentiality {33}

All participant data will be handled in accordance with the EU GDPR and the data protection policies of the University Medical Center Magdeburg. To ensure confidentiality, each participant will be assigned a unique study ID, which will be used for all data collection, storage, and analysis. No directly identifiable personal information (e.g., name, address, date of birth) will be included in the research database. Paper-based documents containing identifiable information (e.g., signed consent forms) will be stored in locked cabinets within secure research facilities accessible only to authorized study personnel. Electronic data, including questionnaire responses, EEG recordings, and physiological data collected via the Labfront app and Garmin Vivosmart 5 tracker, will be encrypted during transfer and stored on secure institutional servers with restricted access. Access to the final pseudonymized dataset will be limited to members of the study team who are directly involved in data processing and analysis. Any publications or presentations arising from the study will use aggregated data only, ensuring that individual participants cannot be identified. Personal data will be retained only for the duration specified in the ethics approval and will be deleted or archived in accordance with institutional and legal requirements.

## Statistical methods

### Statistical methods for primary and secondary outcomes {27a}

All statistical analyses will be performed using RStudio [41, 42] (R Core Team), SPSS ([43]; IBM Corp.), or JASP [44] (https://jasp-stats.org/). Continuous variables will be presented as means ± standard deviation (SD) or median with interquartile range, and categorical data as frequencies and percentages. All tests will be two-sided, with a significance threshold of *p* < 0.05. Effect sizes (Cohen’s *d*, partial *η*^2^, or 95% confidence intervals) will be reported where applicable.

#### Primary analysis

The primary outcome is the change in fatigue severity from baseline (T0) to post-intervention (T1), measured by the MFIS total score. A repeated-measures analysis of variance (ANOVA) or a linear mixed-effects model will be used to test the time x group interaction (active vs. sham taVNS). If baseline group differences occur in relevant covariates (e.g., age, sex, baseline fatigue), these variables will be included as covariates in an analysis of covariance (ANCOVA) framework. Model assumptions (normality of residuals, homoscedasticity) will be assessed visually and analytically. Where appropriate, transformations or alternative models will be considered. Statistical analyses will be conducted blinded to group allocation until the primary analysis is finalized.

#### Secondary analyses

Secondary outcomes include:

- MFIS subscales (physical, cognitive, psychosocial)
- WHOQOL-BREF total and domain scores (quality of life)
- Beck Depression Inventory (BDI-II) (depressive symptoms)
- Epworth Sleepiness Scale (ESS) (daytime sleepiness)
- SDMT (information processing speed)
- tonic and phasic Alertness Task (arousal and vigilance)
- EEG parameters (P50 suppression ratio, rsEEG spectral power)

Each of these outcomes will be analyzed using linear mixed models with fixed effects for time (T0, T1, T2), group (active/sham), and their interaction. Post hoc contrasts will explore within- and between-group changes.

### Who will be included in each analysis

Participants will be included in the respective analyses as follows:

#### Intention-to-treat (ITT) population

The ITT population will include:

- All participants who were randomized
- And who provided at least one post-baseline assessment of the primary outcome (MFIS).

Participants with incomplete data, protocol deviations, or early discontinuation of the intervention will remain in the ITT dataset. Missing values will be handled using maximum likelihood estimation within mixed-effects models.

#### Per-protocol (PP) population

In addition, per-protocol (PP) analyses will be conducted to evaluate the robustness of the results. The PP population will include participants who:

- Completed at least 80% of the prescribed stimulation sessions,
- Had no major protocol violations (e.g., incorrect device use, discontinued medication in violation of stability criteria), and
- Completed both baseline (T0) and post-intervention (T1) assessments.

Participants who were unblinded for safety reasons or who experienced major deviations affecting intervention fidelity will be excluded from the PP analysis.

#### Exploratory analyses

Exploratory analyses (e.g., HRV and activity levels, disease group, baseline fatigue severity, follow-up T2) will include all participants with sufficient available data for the respective model. No imputation will be performed for exploratory outcomes unless explicitly justified.

### How missing data will be handled in the analysis {27c}

All available data will be included in the ITT analysis using maximum likelihood estimation within mixed-effects models, which handle missing values under the assumption of missing at random. Dropout patterns will be examined, and reasons for missingness documented.

### Methods for additional analyses (e.g., subgroup analyses) {27d}

Exploratory analyses will examine potential predictors and moderators of treatment response, including:

- Baseline fatigue severity
- Disease category (neurological, post-infectious)
- Age, sex

Correlations between EEG markers, autonomic measures (HRV), and subjective fatigue improvement will be explored using Pearson or Spearman coefficients, as appropriate. These analyses aim to identify biological response patterns that might predict benefit from taVNS.

### Interim analyses {28b}

No formal interim analyses are planned for this trial as conducting interim analyses could increase the risk of inflated type I error and compromise the integrity of the blinded, randomized design. Moreover, the intervention (taVNS) is a non-invasive, low-risk procedure with a well-established safety profile, reducing the need for early efficacy or safety stopping rules. However, continuous safety monitoring will be conducted throughout the study.

### Protocol and statistical analysis plan {5}

The present document represents the full study protocol and includes a predefined statistical analysis plan for primary, secondary, and exploratory outcomes. Any deviations from this plan will be documented and justified in the final trial report.

## Oversight and monitoring

### Composition of the coordinating center and trial steering committee {3d}

The trial is coordinated at the University Medical Center Magdeburg. The Clinical Trials Unit of the University Medical Center Magdeburg provided support during the planning and preparation phase of the study, including regulatory procedures, study organization, and data management infrastructure.

Given the investigator-initiated, single-center design and the low-risk, non-invasive nature of the intervention, no separate trial steering committee has been established. Overall trial oversight, protocol adherence, and day-to-day trial management are the responsibility of the principal investigator. 

### Composition of the data monitoring committee, its role and reporting structure {28a}

Given the non-invasive nature of taVNS and its established safety profile, no independent data monitoring committee has been constituted. Safety monitoring, data quality control, and protocol compliance will be ensured by the principal investigator in accordance with GCP guidelines and the Declaration of Helsinki.

### Frequency and plans for auditing trial conduct {29}

The trial may be subject to audits by the institutional research quality management of the University Medical Center Magdeburg or by the funding body, if required. Audits may be conducted on request or if indicated. No routine external audits are planned at predefined intervals.

### Protocol amendments {31}

Any substantial amendments to the study protocol (e.g., changes in eligibility criteria, outcomes, or statistical analyses) will be submitted to the Ethics Committee of the University of Magdeburg and updated in the trial registry prior to implementation. All changes will be reported in the final manuscript.

### Dissemination policy {8}

The study results will be submitted for publication in peer-reviewed journals and presented at national and international conferences. Deidentified datasets and statistical analysis scripts will be made available upon reasonable request to the corresponding author after publication.

## Discussion

The present trial aims to evaluate the efficacy of home-based taVNS in reducing fatigue severity across two major diagnostic categories (neurological and post-infectious diseases). taVNS represents a promising candidate intervention because it is non-invasive, safe, and presumably capable of modulating neurobiological pathways implicated in the pathogenesis of fatigue—particularly autonomic regulation, neuroimmune signaling, and cortico-striatal network dynamics. By combining taVNS with continuous physiological monitoring and repeated self-assessments, the study also provides an opportunity to explore potential markers and mechanistic correlates of treatment response.

A key strength of this study is its double-blind, stratified, sham-controlled design, which enhances internal validity and allows rigorous attribution of any observed effects to active vagal stimulation rather than nonspecific device or contextual factors. The inclusion of a broad population across diagnostic boundaries increases both the ecological validity and clinical relevance of the findings and enables exploratory analyses of transdiagnostic or disease-specific response patterns. The use of standardized digital tools (Labfront platform, Garmin Vivosmart 5) further facilitates high-resolution monitoring of adherence, sleep, and autonomic function in participants’ natural environments.

Several challenges should be acknowledged. First, the heterogeneity inherent to a transdiagnostic sample may increase interindividual variability and potentially dilute condition-specific effects. However, the planned stratification, the sample size, and the exploratory subgroup/moderator analyses are designed to mitigate this risk. Second, the limited intervention and follow-up periods restrict conclusions about long-term efficacy or durability of effects; extended follow-up phases may be considered in subsequent studies. Third, although sham stimulation is considered the gold standard in neuromodulation research, subtle differences in perceptibility between active and sham sensations may influence blinding integrity; this will be assessed systematically at the end of the study.

Despite these limitations, the trial is expected to generate valuable evidence on the potential of taVNS as an accessible, home-based treatment for pathological fatigue across chronic diseases. If effective, taVNS could offer a low-risk, scalable intervention with the potential to significantly improve quality of life in a growing population of patients for whom current therapeutic options are insufficient. The transdiagnostic approach employed in this study may also serve as a model for future research targeting shared mechanisms across disease boundaries.

## Trial status

Protocol version: Version: 1.1, Date: 11 February 2026.

Recruitment status: Recruitment activities began on December 08, 2025. However, at the time of manuscript submission, no participant has been enrolled and no written informed consent has yet been obtained. Recruitment is expected to take approximately 24 months.

Estimated completion of follow-up assessments: December 2027.

## Data Availability

No datasets were generated or analyzed during the current study.

## Abbreviations

AE: Adverse event
ANCOVA: Analysis of covariance
ANOVA: Analysis of variance
BDI-II: Beck Depression Inventory (Second Edition)
CE: Conformité Européenne
CRQ: Comfort Rating Questionnaire
DRKS: Deutsches Register Klinischer Studien (German Clinical Trials Register)
EEG: Electroencephalography
EFRE: European Regional Development Fund
eSDMT: electronic Symbol Digit Modalities Test
ESS: Epworth Sleepiness Scale
FSS: Fatigue Severity Scale
fMRI: Functional magnetic resonance imaging
GCP: Good Clinical Practice
GDPR: General Data Protection Regulation
HRV: Heart rate variability
ID: Identification number
IL-6: Interleukin-6
ITT: Intention-to-treat
ME/CFS: Myalgic encephalomyelitis/chronic fatigue syndrome
MFIS: Modified Fatigue Impact Scale
MS: Multiple sclerosis
ON/OFF: Stimulation on/off cycle
PD: Parkinson’s disease
PP: Per-protocol
REDCap: Research Electronic Data Capture
rsEEG: resting-state electroencephalography
SAE: Serious adverse event
SD: Standard deviation
SDMT: Symbol Digit Modalities Test
taVNS: Transcutaneous auricular vagus nerve stimulation
TAP: Test of attentional performance
TNF-α: Tumor necrosis factor alpha
T0: Baseline assessment
T1: Post-intervention assessment
T2: Follow-up assessment
WHOQOL-BREF: World Health Organization Quality of Life – Brief Version
